# Influence of hydrodynamic and functional nonlinearities of blood flow in the cerebral vasculature on cerebral perfusion and autoregulation pressure reserve

**DOI:** 10.1038/s41598-023-32643-z

**Published:** 2023-04-17

**Authors:** Adam Piechna, Krzysztof Cieślicki

**Affiliations:** Institute of Automatic Control and Robotics, Warsaw University of Technology, św. Andrzeja Boboli St. 8, 02-525 Warsaw, Poland

**Keywords:** Computational science, Cerebrovascular disorders

## Abstract

Ensuring the transport of oxygenated blood to the brain is one of the priorities of the human body. In the literature, there are many models of cerebral circulation with different levels of complexity used to assess pathological conditions, support clinical decisions, and learn about the relationships governing cerebral circulation. This paper presents a zero-dimensional cerebral circulation model that considers hydrodynamic nonlinearities and autoregulation mechanisms. The model has been verified using a computational fluid dynamics (CFD) model of the Circle of Willis (CoW) and its supplying and outgoing branches. Despite the considerable simplicity, the presented model captured the dominant features of cerebral circulation and provides good agreement with the CFD model. The errors in relation to the CFD model did not exceed 2.6% and 9.9% for the symmetrical and highly asymmetrical CoW configurations, respectively. The practical application of the model was demonstrated for predicting the autoregulation pressure reserve for different diameters of natural anastomoses: Posterior and Anterior Communicating Arteries. The advantages and limitations of the model were discussed.

## Introduction

The brain is supplied by the carotid and vertebrobasilar systems of vessels interconnected by arterial anastomoses forming, at the base of the brain, the Circle of Willis (CoW). Due to significant anatomical variations of the CoW and many pathologies of the arterial system, a great number of models of cerebral blood flow (CBF) with different levels of complexity are used as cognitive tools to support clinical decisions. In order to accurately reproduce reality, the model should take into account the anatomical, hydrodynamic, and functional conditions of blood circulation. The anatomical conditions are determined by the topography and geometry of the arteries supplying the brain, i.e. the bilateral internal carotid system and the vertebrobasilar system. The system’s topology describes how the anastomoses connect both circulatory systems (the type of the CoW). The geometric structure of the arteries provides information about their shape and dimensions. The hydrodynamic conditions are a derivative of the tortuosity of the arteries and their multiple divisions and connections, which cause the flow disturbances and, in consequence, a nonlinear pressure-flow relationship. The functional conditions result from the active intrinsic ability of the cerebral vascular bed to maintain constant CBF in a specific range of systemic pressure changes. Macroscopically it manifests by the nonlinear and non-monotonic dependence of global peripheral vascular resistance on the value of mean arterial pressure.

Many different models of cerebral blood circulation have been proposed in the literature starting from the simplest, linear, steady-state 0D models where the Hagen-Poiseuille (H-P) formula was used to calculate arterial resistances (Hillen^1^, Cassot^2^, Cassot^3^). Cieślicki and Cieśla^4^ compared the flow results obtained from a unique physical model of the cerebral arterial circle made of Plexiglas with the non-stationary and nonlinear 0D numerical model. They take into account the arterial tortuosity and the development of a velocity profile in short arteries. The more complicated unsteady 1D models took into account the elasticity of arterial walls and the inertial effects of flow (Hillen^5^, Kufahl and Clark^6^, Raines^7^). In recent years, the use of CFD to model flow in actual geometries derived from medical imaging has become prevalent (Saqr et al.^8^, Jung-Jae Kim et al.^9^, Kwang-Chun Cho et al.^10^, Piechna and Cieślicki^11^ or Xiang et al.^12^). While the last concept is very attractive, its credibility and accuracy depend, in particular, on the accuracy of the geometry recreation, applied boundary conditions, and the adopted autoregulation models. Another limitation of the CFD models is their inherent feature: the relatively long computation time that limits the modeling of many variants.

One of the visible present trends of the research in question is an attempt to combine 1D and 3D models. The latter is used to define velocity, pressure, and stress fields in the local section of the circulatory system and the former to define the boundary condition for pressure/flow at its inlet/outlet. An example could be the works of Passerini et al.^13^ and Blanco et al.^14^.

Data obtained from computer simulations can also be used for machine learning of physician support systems. Rutkowski et al.^15^ used data from accurate CFD analyses to augment data from cerebral four-dimensional flow phase-contrast magnetic resonance imaging (4D flow MRI) with the use of a convolutional neural network. This makes it possible to quickly obtain a highly accurate physiological flow field based on MRI-derived velocity fields.

We present only a short review of the computational models of cerebral circulation, highlighting the most important factors. Readers interested in a more in-depth review are referred for example to the valuable article by Liu et al.^16^. One of the important conclusions is that the balance between computational complexity and physiological accuracy is still an open question.

The model proposed in this work captures the dominant features of cerebral circulation, is relatively simple, and provides fast solutions suitable for real-time clinical decisions. Two phenomenological formulas were applied to estimate the nonlinear resistance of afferent segments^4,17^ which required the specification of only the basic dimensions of an individual segment, such as the diameter, length, and bend curvature radius. The obtained results were confronted with the 3D numerical model based on the Finite Volume Method used to solve Navier–Stokes equations. The model was extended by the autoregulation model, and the cerebral autoregulation pressure reserve was investigated in different occlusion scenarios. The influence of hydrodynamic nonlinearities of blood flow in the cerebral vasculature was investigated and the value of blood pressure at the level of the CoW, which determines the cerebral perfusion to the anterior, middle, and posterior parts of the brain, was estimated. An additional motivation of this study was to evaluate the usability of this relatively simple model compared with the CFD models and assess its advantages and drawbacks.

## Results

### Model validation

Calculations of pressures and flows in the reference CoW without an autoregulation mechanism were performed using a 0D linear and nonlinear model and a full 3D CFD simulation. Analogous calculations were carried out for several representative pathologies of the cerebral circulation associated with complete occlusion of the supplying arteries.

**Figure 1:**
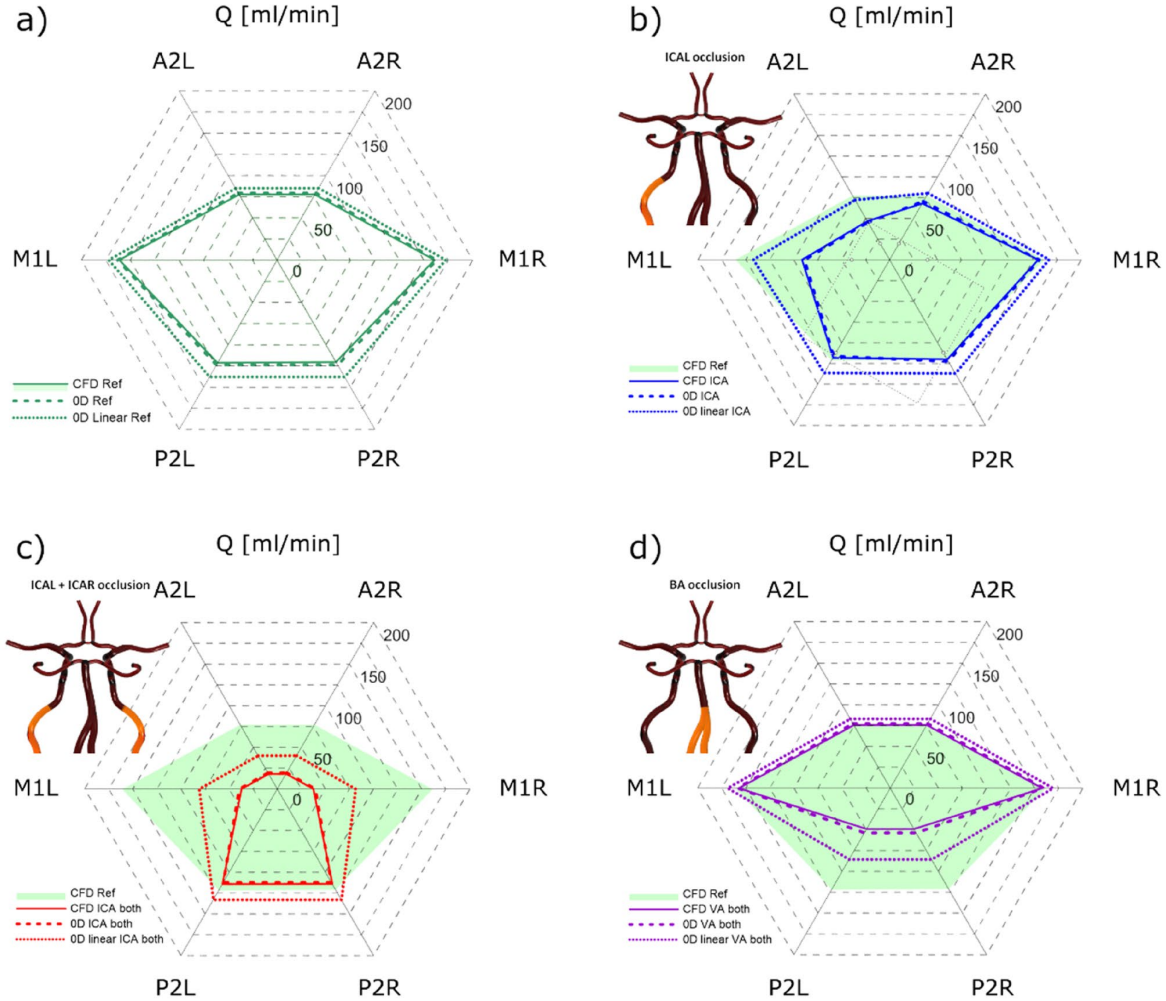
(**a**) A comparison of the flow rates in branches of the reference (physiological) CoW between the CFD model and the 0D models. (**b**–**d**) A comparison of the flow rates in the CoW branches for the CFD (solid line) and 0D model, nonlinear (dashed line) or linear (dotted line) for different occlusion of supplying arteries configurations (shown as infographics). The green area denotes the CoW without any occlusions.

Figure 1a shows a comparison of the CFD model and the 0D model with nonlinear or linear resistances of the arterial segments. In Fig. 1b–d a comparison between a CFD model and 0D model was presented for several pathological configurations of the CoW.

The results were also summarized in Supplementary Table 1 in Appendix A.

### Autoregulation pressure reserve

To show the importance of the modeling of the hydrodynamic nonlinearities and to demonstrate the capabilities of the proposed model, the gradually increasing occlusion of both ICAs was simulated to test the autoregulation pressure reserve (APR).

The APR determines a safe drop or increase in systemic pressure while maintaining a constant cerebral flow. For the purposes of this work, it was defined as the difference in arterial pressure at the level of the arteries of the base of the brain and the lower or upper limit of autoregulation.

$$APR=\left\{\begin{array}{c}{P}_{{cerebral\,artery}^{*}}-{P}_{lower\,limit\,of\,autoregulation}\\ {P}_{upper\,limit\,of\,autoregulation}-{P}_{{cerebral\,artery}^{*}}\end{array}\right\}$$

*—pressure at the level of the major cerebral arteries at the base of the brain.

The APR then takes two values. If both are positive, cerebral flow is preserved. When one is negative, it means an exit from the APR by too low or too high pressure, respectively. Although the APR value is not clinically available, it is of great importance in determining the hemodynamic status of patients.

**Figure 2:**
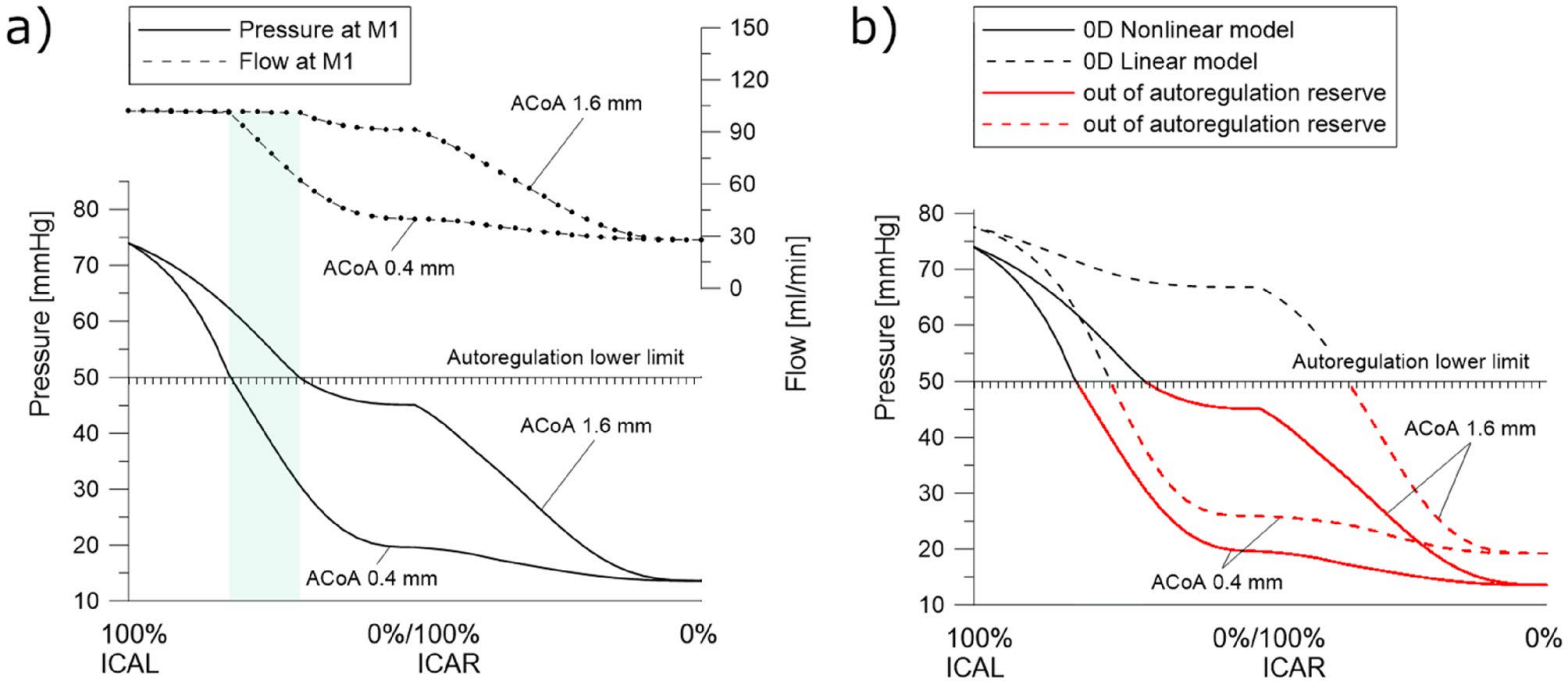
(**a**) Pressure (solid lines) and flow rates (dashed line) in the middle cerebral artery for the gradually increasing occlusion of both ICAs. (**b**) Comparison of autoregulation reserve for nonlinear (solid lines) and linear (dashed lines) models.

**Figure 3:**
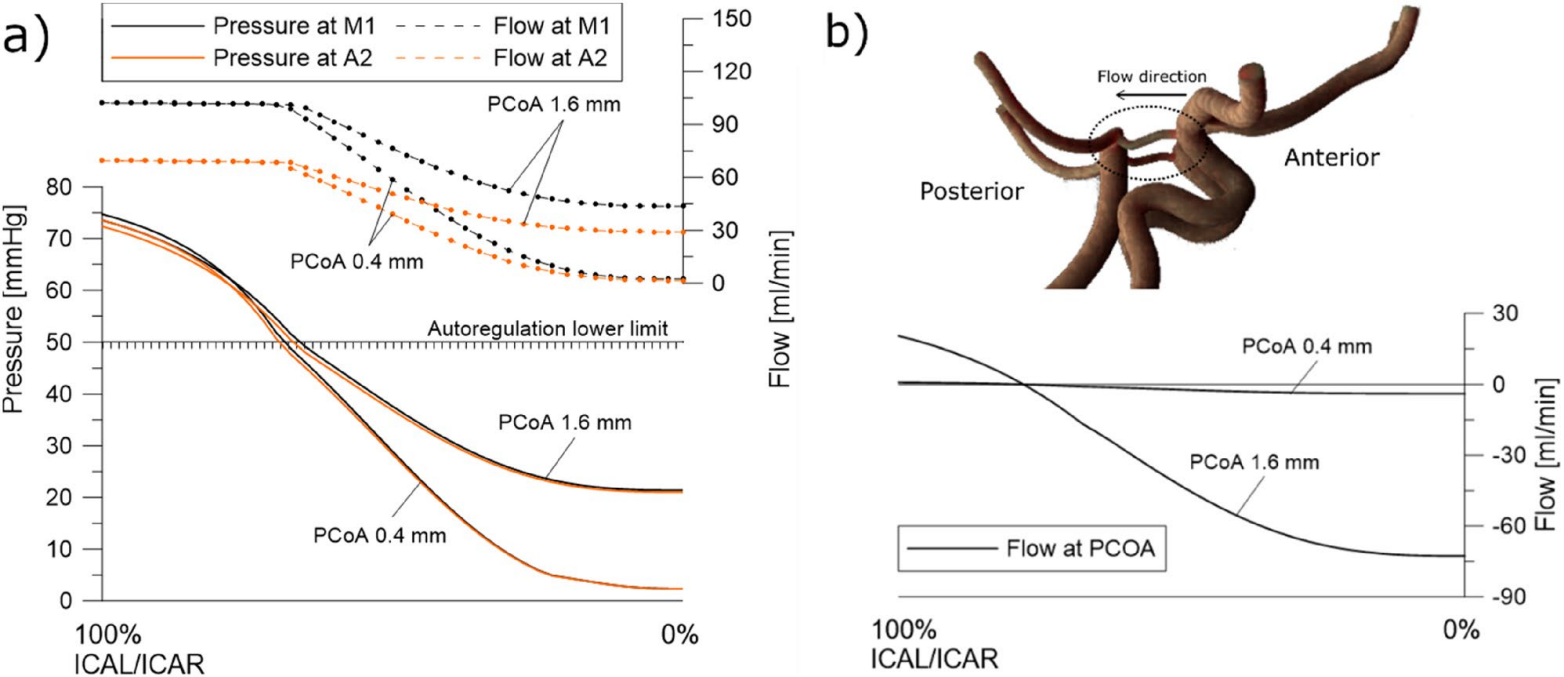
(**a**) Pressure (solid lines) and flow rates (dashed line) in the middle and anterior cerebral artery for concurrent occlusion of both ICAs. (**b**) Flow rates in the posterior communicating arteries during the concurrent occlusion of both ICAs. A flow direction denoting positive flow rates was schematically shown using an arrow.

Two 0D models were compared: a linear model and a model with hydrodynamic nonlinearities. Both models have an autoregulation mechanism implemented. Also, an ACoA and PCoAs diameters impact was investigated as one of the main factors that determine blood flow redistribution. The obtained results are presented in Figs. 2 and 3.

## Discussion

As can be seen in Fig. 1 in the linear model, cerebral flows are higher than in the CFD simulation. The maximum relative error between the linear model and the CFD model in the physiological situation was 14.4%. After applying nonlinear resistances of the arterial segments calculating according to formulas (1) and (2) our model fitted the numerical model extremely well with a maximum error of 2.6%. However, the error becomes higher when pathologic situations are studied. For a completely blocked BA, the error increased to 74.8% and 9.9% for the linear and non-linear model, respectively.

**Figure 4:**
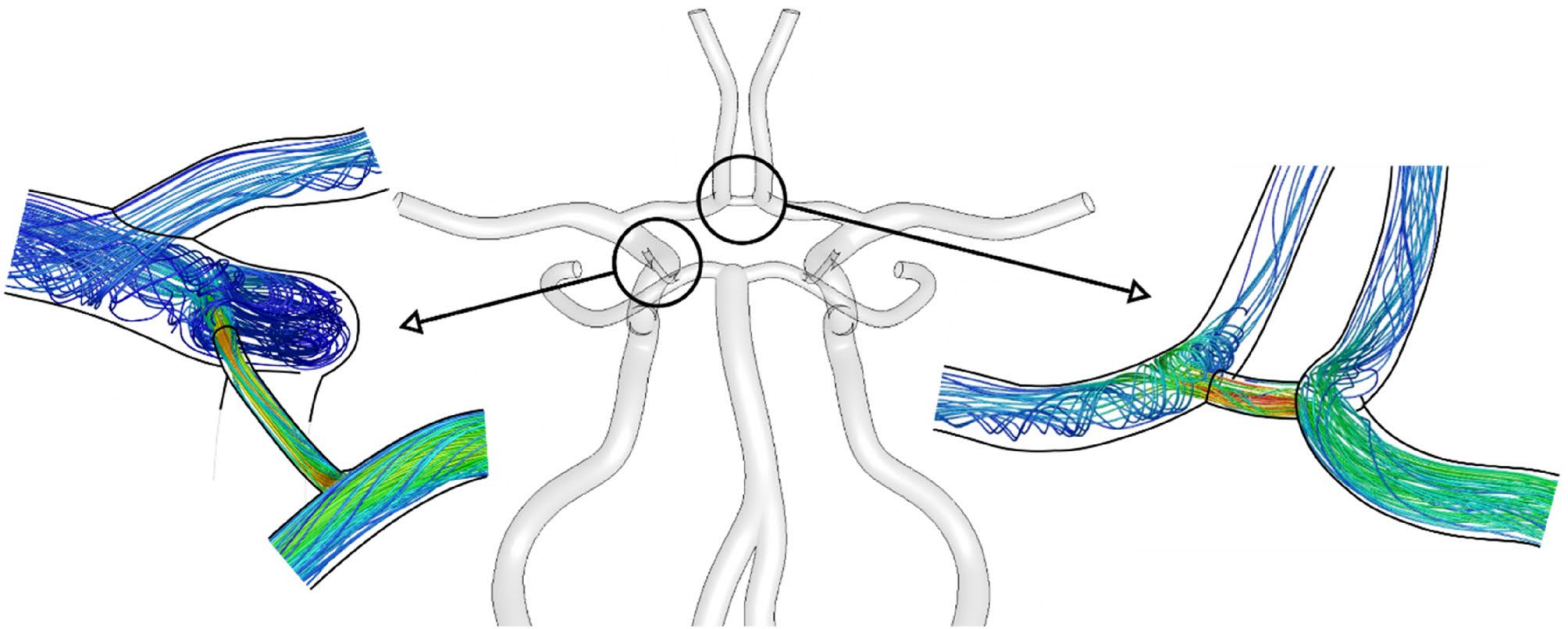
Streamlines showing the additional recirculation areas and impinging jets in junctions.

As is commonly known, when one or more of the supplying arteries is blocked, the flow rate in the communicating arteries increases. Therefore, to understand the possible source of the lower accuracy of our model in these cases we investigated the streamlines of the flow in the vicinity of nodes with communicating arteries. As we can see in Fig. 4 in these regions the additional recirculation areas and impinging jets appear.

The importance of nonlinearities resulting from the development of the velocity profile is then increased by an additional effect that has not been taken into account in formula (2): energy losses in nodes. In the literature, a number of empirical formulas for estimating the pressure drop in junctions can be found. However, in most cases, they are valid in turbulent flow regimes. They also depend on many parameters: relative channel diameters, connection angle, flow rate ratios, and flow direction. The proposed model could be easily extended to take into account the additional junction resistance. However, in practical applications, this is a secondary element, because most cases of clinical significance are less drastic than the tested examples of complete arterial blockage, and the relative error of the model will be in the order of a few percent. Also, a limitation is the higher number of parameters that have to be calibrated and the limited validity of the equations.

The second source of non-linearity included in our simulations was arterial tortuosity which generates the secondary flow in the planes perpendicular to the main axis of arteries and causes additional dissipation of energy.

**Figure 5:**
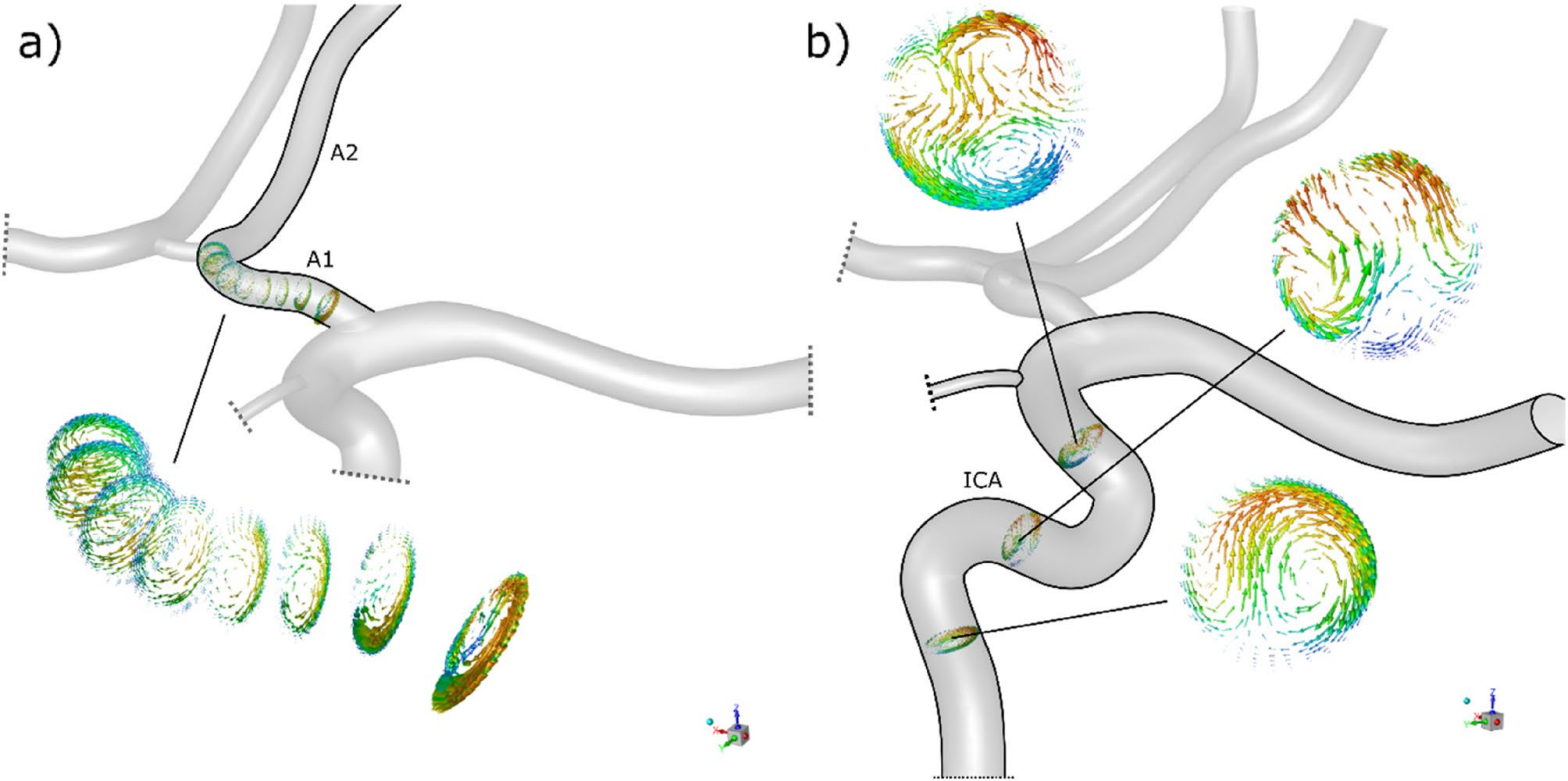
Velocity vectors in planes perpendicular to the axis of the arteries represent secondary flows due to (**a**) rapid change of flow direction and (**b**) vessel curvature.

In Fig. 5, velocity vectors of secondary flows taken from the CFD calculation of the reference CoW are shown in the artery with a high curvature and just after a bifurcation where there is a rapid change in flow direction.

The secondary flows show characteristic transverse vortices. Interestingly, in the case of a single bend, there is a pair of transverse vortices well known in the literature. Behind successive bends, the structure is more complicated, and more vortices can be formed. Cieślicki and Piechna thoroughly investigated the topic in^17^. An interesting observation is that despite completely different velocity fields in spatially bent channels and, for example, coil-shaped or sinusoidal channels, the resistance characteristics are the same and can be described by the formula used in the presented model (1).

To date, we have validated the proposed model and estimated the range of its possible inaccuracy. As an example of the practical usage of the model, we conducted a simulation to investigate the autoregulation pressure reserve. The progressive occlusion of the left carotid artery was modeled. Its complete closure is followed by the occlusion of the right artery on the opposite side. Until the pressure is higher than the lower limit of autoregulation, the peripheral arteries dilate, reducing resistance, and flow is preserved. Once the lower limit is exceeded, the peripheral arteries cannot expand further, and flow is reduced. In Fig. 2a, we present the values of flows and pressures for the nonlinear model for two diameters of the anterior communicating artery, acting as an additional channel to maintain cerebral flows when the carotid arteries are asymmetrically occluded. For small ACoA (0.4 mm), exit from autoregulation occurs as early as 34.1% stenosis (calculated from diameter change) of the left ICA and results in an abrupt drop in the left middle cerebral artery (M1) flow rate. For large ACoA (1.6 mm), autoregulation fails after reaching 58.5% stenosis, and the following flow rate reduction is much smaller. In patients with unilateral carotid artery stenosis, a thin ACoA is an indicator of an increased risk of hypoxia. Figure 2b summarizes the pressure plots for the same scenario recalculated with a linear model. Failure to account for nonlinearities in the cerebral circulation model can lead to misleading hypotheses. As can be seen, the autoregulation reserve in the linear model is far more optimistic, completely changing the critical value of ICA stenosis. For thick ACoA, with complete occlusion of the left ICA, the system is still within the pressure range of the autoregulation area. With bilateral carotid artery stenosis and symmetric CoW, the diameter of the ACoA ceases to matter, and the values of pressures and flow rates are the same regardless of its diameter. In such a scenario, the PCoA diameters start to matter. Figure 3 shows the scenario of simultaneous stenosis of both carotid arteries. In this case, the posterior communicating arteries enabling flow from the posterior to the anterior part of the CoW have an essential role. However, we observe a different pattern. The exit from autoregulation for PCoAs of different thicknesses occurs practically for the same value of arterial stenosis, while the subsequent decrease in flow is much greater for thin PCoAs. In this case, the dilatation of the peripheral arteries takes all the brunt at the onset of arterial occlusion, with a significant role of the PCoA after leaving the autoregulation range.

In the presented study, no direct comparison was made with empirical studies, as this was not the purpose of the work. One should be aware that model errors resulting from the difficulty of determining, for example, autoregulation parameters, the accuracy of recreating geometry of small arteries, or blood properties for a specific patient can be significant. This is a feature of most developed numerical models. In complex models where more detailed parameters must be specified, the model’s accuracy may paradoxically be lower than in simpler models.

## Conclusions

A fast and easy-to-implement 0D model that takes into account the hydrodynamic and functional nonlinearities was proposed and validated with an accurate but time-expensive CFD simulation. Despite its simplicity, the model has high accuracy (2.6% relative error for symmetrical CoW and up to 9.9% for highly pathological CoW). We believe that such a model shows high level of usefulness resulting mainly from its speed and relatively simple definition—using parameters that are easy to obtain, e.g. from medical imaging data. A practical usage of the model was demonstrated to simulate different scenarios of the CoW occlusions. A different pattern was observed for the gradually increasing occlusion of the ICAs. The obtained results also indicated that the autoregulation mechanisms, whose task is to maintain a constant cerebral flow, paradoxically favor greater pressure drops at the Circle of Willis level. As a result, the closure of a large supply artery, such as the internal carotid artery, quickly depletes the autoregulation pressure reserve.

Also, the article shows that the nonlinearities of the vascular system caused by the autoregulation mechanisms and the hydrodynamic phenomena in the supplying and cerebral arteries, as well as the state of the anastomoses of the Circle of Willis, significantly affect the APR, particularly in the event of the pathological reduction of the blood supply. We found that linear methods are not applicable at all to modeling the intracranial circulation. The nonlinear effects are particularly pronounced in conditions of pathological occlusion of the supplying arteries.

The main conclusion is that simple models based on the 0D approach could be an interesting alternative or could complement other, more complicated methods. Its advantages and benefits are: low computational cost, simple model definition, and a small number of parameters while achieving good accuracy.

## Material and methods

### Rationale for model simplification and model assumptions

As the main motivation of the research was to propose a relatively simple but accurate model, we had to choose which phenomena are important and should be taken into account and which can be disregarded.

The Reynolds (*Re*) number in the main arteries of cerebral circulation is in the range of 100–500. As the critical *Re* value for the pulsatile blood flow in bended canals with lots of branches is not well defined, a common agreement is that cerebral flow is laminar. The resistance of the straight and long arteries can be described by the Hagen-Poiseuille formula, and for bended and short arteries, an empirical formula can be found in the literature.

In the presented model, we decided to treat blood as a Newtonian fluid. It is a common agreement that in such caliber of arteries taking into account the non-newtonian properties of the blood has a negligible impact on pressure drop and flow rates evaluation^18,19^. Blood rheology is important in estimating local shear stresses, especially in areas of increased flow velocity, such as arterial stenosis^19^, or in areas of recirculation, such as aneurysms^8^, which does not apply to the proposed model. Nevertheless, to estimate the error introduced, a comparative simulation of flow through the arterial circle was performed for a constant value of the dynamic viscosity coefficient and for the Carreau-Yasuda (C-Y) model. In the C-Y model, the stress dependence of viscosity is given by the relation:

1 $$\eta \left(\dot{\gamma }\right)={\eta }_{\infty }+\left({\eta }_{0}-{\eta }_{\infty }\right){\left(1+{(\lambda \dot{\gamma })}^{a}\right)}^{\left(n-1\right)/a}$$

where: $\eta$—dynamic viscosity [Pa*s], $\dot{\gamma }$—shear strain rate [1/s].

for model parameters we take: ${\eta }_{0}$ = 0.16 Pa s, ${\eta }_{\infty }$ = 0.0035 Pa s, $\lambda$ = 8.2 s, $a$ = 0.64, $n$ = 0.2128.

The obtained differences in flow rates prediction between two models were below 0.1%.

To simplify the model as much as possible and to limit the number of parameters that need to be identified, we decided to model the cerebral circulation in a stationary manner. Hillen et al.^1,5^ compared the stationary and unsteady models of the 0D cerebral circulation and found no differences in the distribution of blood flow and less than 10% error in the flow rate values. Given that this assumption introduces a significant error, we continue to maintain that the advantages of keeping the model as simple as possible dominates.

One of the key factors to be addressed is the outlet boundary conditions. As was shown in^17^, an autoregulation mechanism needs to be taken into account in cerebral circulation modelling. A simple assumption of lumped resistance or Windkessel (in unsteady simulation) boundary condition could lead not only to quantitatively but also qualitatively incorrect results. In the presented model, we introduce a simple autoregulation model defined by a few parameters. It should be noted that obtaining patient-specific autoregulation parameters is a major challenge.

### 0D model

**Figure 6:**
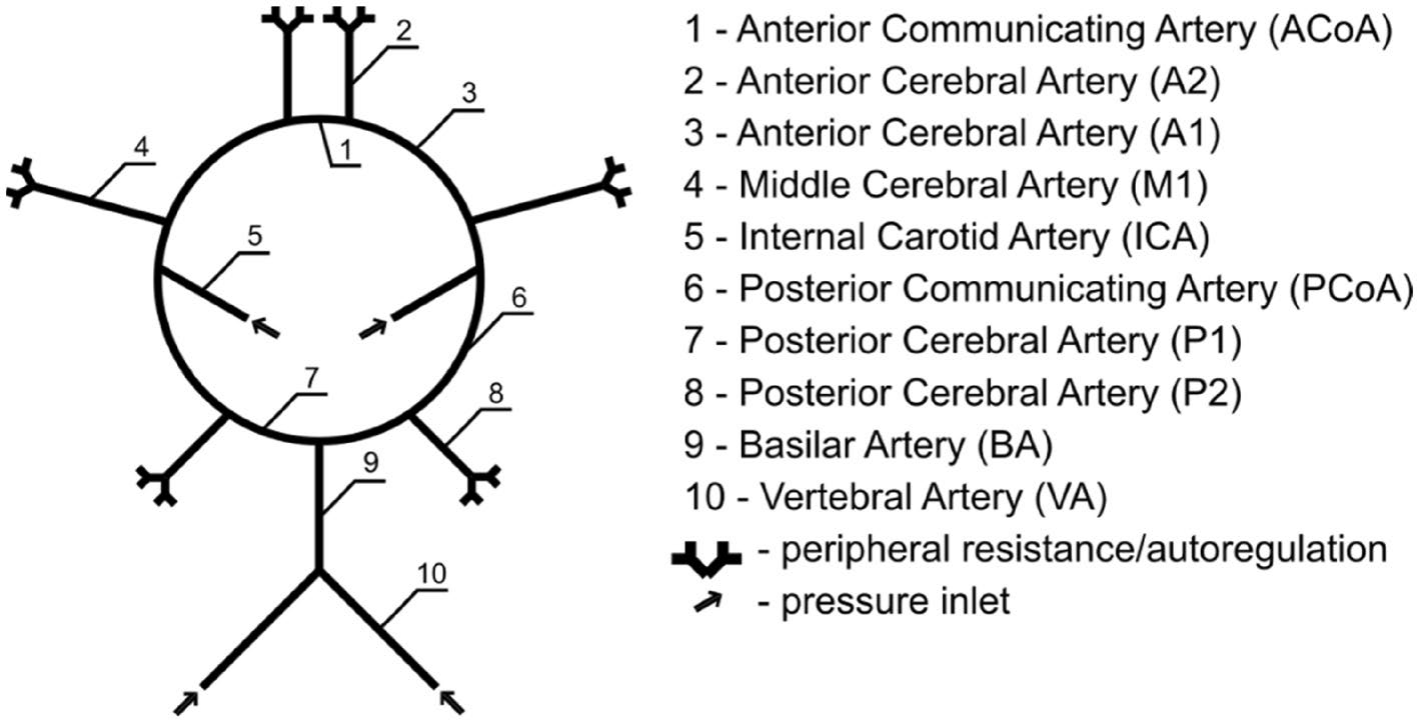
A nonlinear 0D 18-element model of the CoW.

The developed model is an extension of the analytic model of the flow in the CoW based on the lumped hydraulic resistances. An 18-element model of the CoW was constructed consisting of nonlinear resistances (Fig. 6).

Two sources of nonlinearities were taken into account: one coming from the curvature of the vessels and the other from the velocity profile development. To describe them, semi-empirical formulas were used in which the nonlinear effects were included in additive correction terms. The empirical formula for the normalized hydraulic resistance of tortuous segments developed by the authors in previous studies^17^ is as follows:

2 $$\frac{{R}_{t}}{{R}_{HP}}=0.526+\sqrt{0.225+0.022\sqrt{\frac{d}{{a}_{k}}}Re}$$

where: ${R}_{t}$—hydraulic resistance of tortuous artery [mmHg min/ml], ${R}_{HP}$—hydraulic resistance from Hagen-Poiseuille formula [mmHg min/ml], $d$—artery diameter [m], ${a}_{k}$—artery curvature radius [m], $Re$—Reynolds number (based on the average velocity and artery diameter).

Note that for very large curvature radius, the equation tends toward the Hagen-Poiseuille formula. In turn, the dynamic hydraulic resistance of the short segments was calculated from a formula described in the literature^4^:

3 $$\frac{{R}_{l}}{{R}_{HP}}=1+0.044\frac{d}{l}Re$$

where: ${R}_{l}$—hydraulic resistance of short segment of the artery [mmHg min/ml], $a$—artery diameter [m], $l$—artery length [m].

To solve the equations, the node potential method was used. As classical nodal analysis assumes that resistances are constant, a numerical procedure was implemented to iteratively adjust the resistance values until a set convergence level was obtained. The procedure was performed in the Java programming language with the help of the EJML library for solving linear equations. All derived equations are presented in the Appendix B together with model diagram (Supplementary Fig. 1).

### CFD model

**Figure 7:**
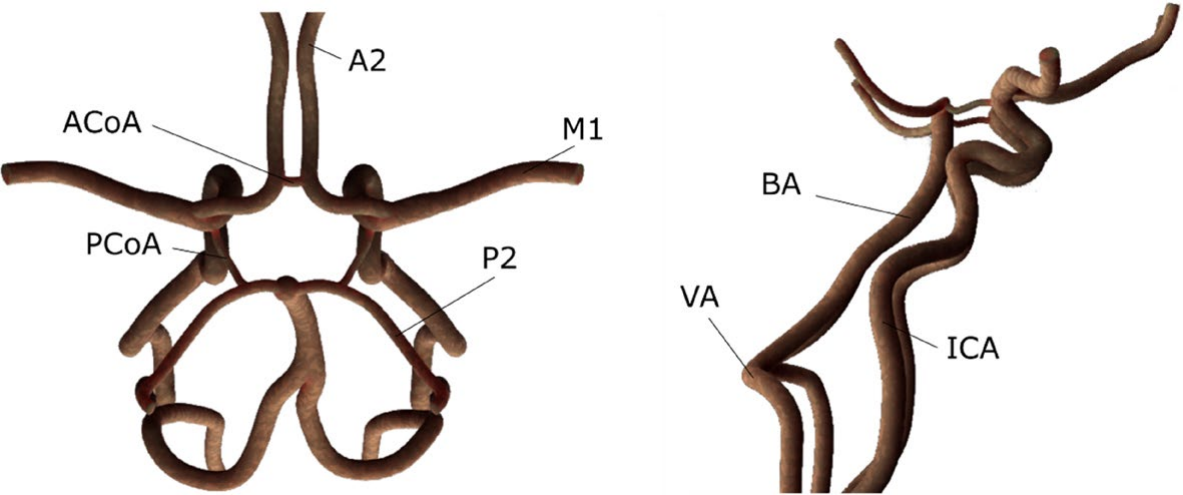
Two projections/views of a modeled CoW.

Arterial topography, as shown in two projections in Fig. 7, was recreated from the Nowiński’s Brain Atlas^20^ using the Space Claim Direct Modeller CAD software.

The diameters, length and curvature radius of the arteries are presented in Table 1.

**Table 1:** Dimensions of the analyzed model of the CoW.

Segment	Length [mm]	Diameter [mm]	Curvature radius [mm]	Formula used
A2L	32.37	2.20	15.00	(1)
A2R	32.37	2.20	15.00	(1)
A1L	13.17	2.20	5.00	(2)
A1R	13.17	2.20	5.00	(2)
M1L	30.78	2.70	12.50	(1)
M1R	30.78	2.70	12.50	(1)
ICAL	250.00	4.00	10.00	(1)
ICAR	250.00	4.00	10.00	(1)
PCoAL	10.30	0.87	5.00	(2)
PCoAR	10.30	0.87	5.00	(2)
VAL	130.00	3.70	35.00	(1)
VAR	130.00	3.70	35.00	(1)
P2L	29.07	1.90	12.00	(1)
P2R	29.07	1.90	12.00	(1)
P1L	7.23	1.90	5.00	(2)
P1R	7.23	1.90	5.00	(2)
ACoA	5.00	1.20	40.00	(2)
BA	23.00	4.00	25.00	(1)

The geometry was discretized using the ANSYS Meshing software. A tetrahedral mesh with prism layers was generated. In the next step, a conversion to polyhedral mesh was performed in the ANSYS Fluent software. A three meshes with different cell counts were generated, and a mesh independence test was performed. An approximate length scaling factor between meshes was 1.3. Two scenarios were investigated: a blood flow in a reference CoW and in CoW with left ICA blocked. A flow rate in left M1 was defined as an observed value. Percentage difference and Grid Convergence Index (GCI) based on generalized Richardson Extrapolation^21^ of observed value was calculated and presented in Table 2.

**Table 2:** Mesh convergence study results.

Mesh	Mesh elements	Reference CoW			ICAL blocked		
		Flow rate at M1L [ml/min]	% difference	GCI index	Flow rate at M1L [ml/min]	% difference	GCI index
1	725,910	161,10	0,54%	0,0075%	91,61	0,66%	0,77%
2	1,506,684	161,98	0,01%	0,0001%	92,22	0,32%	0,37%
3	2,801,997	161,99	–	–	92,52	–	–

Obtained differences between models are very small with slightly larger values for asymmetric CoW case. Finally, a mesh with 0.7 million polyhedral cells was used for further simulations.

Flow simulations were completed using the ANSYS Fluent software which makes use of the finite volume method to solve the Navier–Stokes equations. The SIMPLE algorithm for pressure–velocity coupling was used. The second-order spatial discretization method was used for the momentum equations. Convergence was monitored by observing the residuals of the equations and flow rates on the model outlets: namely anterior, middle, and posterior arteries of the CoW. A pressure inlet boundary condition was applied with a constant value of 93 mmHg.

On the outlet boundaries, i.e. the distal ends of the CoW branches, peripheral resistances were applied to mimic the effects of arterial trees in the anterior, middle, and posterior parts of the brain. All applied boundary conditions are summarized in Table 3.

**Table 3:** Applied boundary conditions.

	Boundary type	Pressure [mmHg]	Resistance [Pas/m^3^]
ICA	Pressure inlet	93	–
VA	Pressure inlet	93	–
P2	Pressure outlet with resistance	10	4.22e9
M1	Pressure outlet with resistance	10	3.52e9
A2	Pressure outlet with resistance	10	7.04e9

As the resistance outlet condition was not available in the software used, a short additional procedure was written in the C programming language. On the arterial walls, a no-slip condition was imposed.

### Model extension

**Figure 8:**
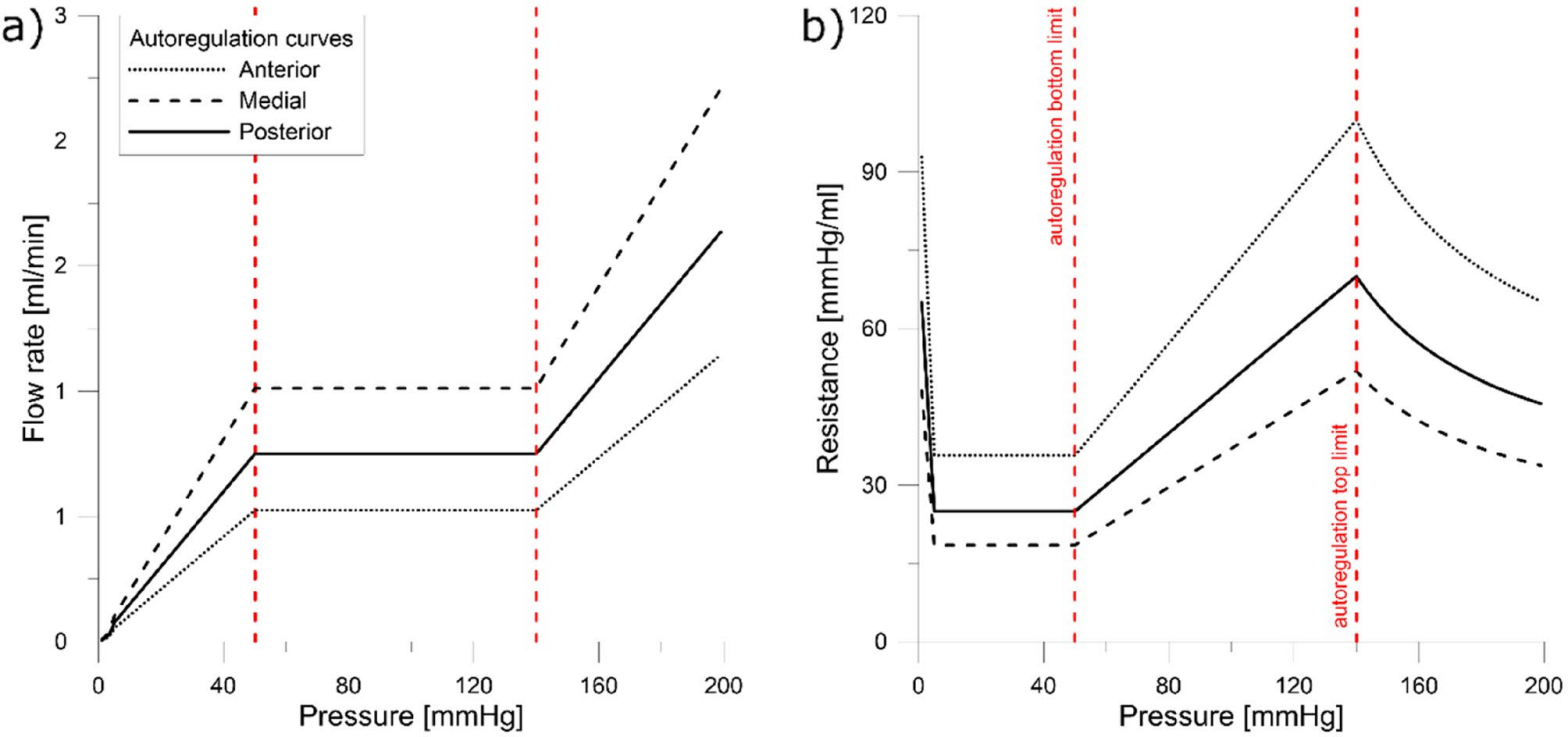
Model of ideal autoregulation of terminal resistances.

To show the usability and extension possibilities, an autoregulation mechanism was implemented in the presented model as it is crucial for accurate cerebral blood flow distribution modeling^11^. To include the functional conditions, the mechanism of autoregulation of terminal resistances and the pressure-resistance characteristics presented in Fig. 8 were applied.

The bottom and the upper limit of autoregulation were highlighted with red dashed lines and were the same for each part of the CoW: 50 mmHg and 150 mmHg, respectively. The values of termination resistances for each part of the CoW are summarized in Table 4.

**Table 4:** Autoregulation model parameters.

CoW part	Mean blood flow [ml/min]	Minimum resistance [mmHg min/ml]	Maximum resistance [mmHg min/ml]
Anterior	111 * 2	0.46	1.31
Midle	162 * 2	0.32	0.90
Posterior	120 * 2	0.44	1.20

## Supplementary Information

Supplementary Information 1.

Supplementary Information 2.

## Data Availability

The synthetic datasets generated and analysed during the current study are available in the supplementary materials. Full dataset and simulation input files are available from the corresponding author upon reasonable request.
