# Supplementary material for: Influence of hydrodynamic and functional nonlinearities of blood flow in the cerebral vasculature on cerebral perfusion and autoregulation pressure reserve
Source: Sci Rep. 2023 Apr 17;13:6229. doi: 10.1038/s41598-023-32643-z (PMC10110590; doi:10.1038/s41598-023-32643-z)
Supplement: Supplementary file 1 — Supplementary Information 1. [file 41598_2023_32643_MOESM1_ESM.docx]

**Appendix A**

Supplementary Table 1. A comparison of the flow rates in branches of the reference (physiological) CoW and for different occlusion of supplying arteries configurations between the CFD model and the 0D models.

|  |  | Ref | | ICAL blocked | | Both ICA blocked | | Both VA blocked | |
| --- | --- | --- | --- | --- | --- | --- | --- | --- | --- |
| A2R | CFD | 77,3943 |  | 67,7949 |  | 18,023 |  | 75,8541 |  |
|  | 0D Lin | 84,8051 | 9,6% | 80,0017 | 18,0% | 39,9969 | 121,9% | 83,2176 | 9,7% |
|  | 0D NL | 79,3018 | 2,5% | 70,6326 | 4,2% | 19,4395 | 7,9% | 77,3597 | 2,0% |
| M1R | CFD | 161,181 |  | 154,805 |  | 37,2945 |  | 157,749 |  |
|  | 0D Lin | 172,247 | 6,9% | 166,306 | 7,4% | 81,2372 | 117,8% | 169,022 | 7,1% |
|  | 0D NL | 163,11 | 1,2% | 156,503 | 1,1% | 39,5921 | 6,2% | 159,062 | 0,8% |
| P2R | CFD | 120,705 |  | 120,132 |  | 114,119 |  | 48,9942 |  |
|  | 0D Lin | 138,11 | 14,4% | 137,385 | 14,4% | 133,023 | 16,6% | 85,6228 | 74,8% |
|  | 0D NL | 123,944 | 2,7% | 122,364 | 1,9% | 111,998 | -1,9% | 53,8499 | 9,9% |
| P2L | CFD | 121,535 |  | 118,292 |  | 114,317 |  | 49,0208 |  |
|  | 0D Lin | 138,11 | 13,6% | 136,823 | 15,7% | 133,023 | 16,4% | 85,6228 | 74,7% |
|  | 0D NL | 123,944 | 2,0% | 116,114 | -1,8% | 111,998 | -2,0% | 53,8499 | 9,9% |
| M1L | CFD | 161,099 |  | 91,6108 |  | 37,3053 |  | 157,344 |  |
|  | 0D Lin | 172,247 | 6,9% | 142,172 | 55,2% | 81,2372 | 117,8% | 169,022 | 7,4% |
|  | 0D NL | 163,11 | 1,2% | 88,6249 | -3,3% | 39,5921 | 6,1% | 159,062 | 1,1% |
| A2L | CFD | 77,4022 |  | 46,6095 |  | 18,0532 |  | 75,778 |  |
|  | 0D Lin | 84,8051 | 9,6% | 71,8765 | 54,2% | 39,9969 | 121,6% | 83,2176 | 9,8% |
|  | 0D NL | 79,3018 | 2,5% | 45,2388 | -2,9% | 19,4395 | 7,7% | 77,3597 | 2,1% |
|  |  | [ml/min] |  | [ml/min] |  | [ml/min] |  | [ml/min] |  |
