# Supplementary material for: Influence of hydrodynamic and functional nonlinearities of blood flow in the cerebral vasculature on cerebral perfusion and autoregulation pressure reserve
Source: Sci Rep. 2023 Apr 17;13:6229. doi: 10.1038/s41598-023-32643-z (PMC10110590; doi:10.1038/s41598-023-32643-z)
Supplement: Supplementary file 2 — Supplementary Information 2. [file 41598_2023_32643_MOESM2_ESM.docx]

**Appendix B**

**
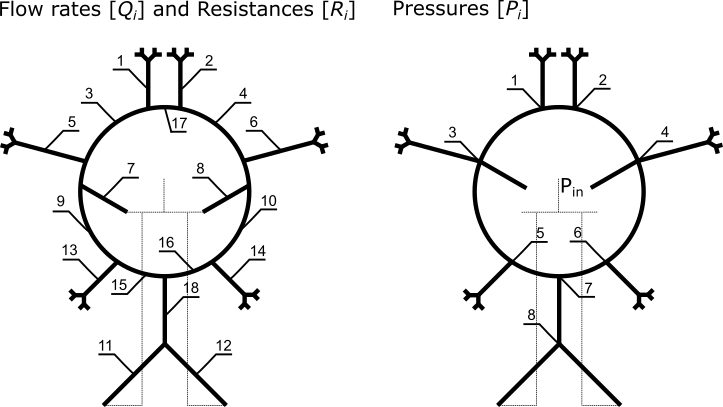
**

Supplementary Figure 1. 0D Model diagram with node and branch numbers

0D model equation set:

| $Q_{3}-Q_{1}-Q_{17}=0$ | $\frac{P_{3}-P_{1}}{R_{3}}-\frac{P_{1}}{R_{1}}-\frac{P_{1}-P_{2}}{R_{17}}=0$ |
| --- | --- |
| $Q_{4}-Q_{2}+Q_{17}=0$ | $\frac{P_{4}-P_{2}}{R_{4}}-\frac{P_{2}}{R_{2}}-\frac{P_{1}-P_{2}}{R_{17}}=0$ |
| $Q_{7}-Q_{3}-Q_{5}-Q_{9}=0$ | $\frac{P_{in}-P_{3}}{R_{7}}-\frac{P_{3}-P_{1}}{R_{3}}-\frac{P_{3}}{R_{5}}-\frac{P_{3}-P_{5}}{R_{9}}=0$ |
| $Q_{8}-Q_{4}-Q_{6}-Q_{10}=0$ | $\frac{P_{3}-P_{1}}{R_{3}}-\frac{P_{1}}{R_{1}}-\frac{P_{1}-P_{2}}{R_{17}}=0$ |
| $Q_{15}-Q_{13}+Q_{9}=0$ | $\frac{P_{3}-P_{1}}{R_{3}}-\frac{P_{1}}{R_{1}}-\frac{P_{1}-P_{2}}{R_{17}}=0$ |
| $Q_{16}-Q_{14}+Q_{10}=0$ | $\frac{P_{3}-P_{1}}{R_{3}}-\frac{P_{1}}{R_{1}}-\frac{P_{1}-P_{2}}{R_{17}}=0$ |
| $Q_{18}-Q_{15}-Q_{16}=0$ | $\frac{P_{3}-P_{1}}{R_{3}}-\frac{P_{1}}{R_{1}}-\frac{P_{1}-P_{2}}{R_{17}}=0$ |
| $Q_{11}+Q_{12}-Q_{18}=0$ | $\frac{P_{in}-P_{8}}{R_{11}}-\frac{P_{in}-P_{8}}{R_{12}}-\frac{P_{8}-P_{7}}{R_{18}}=0$ |

where:

$R_{1},R_{2},R_{5},R_{6},R_{13},R_{14}$ are sum of resistance of the corresponding cerebral artery and attached resistance of the arterial network (values given in table 3 and 4 in the main text, for model without and with autoregulation mechanism).

For linear model resistances of the cerebral arteries were computed from Hagen-Poiseuille formula:

$R_{HP}=\frac{128 \mu L}{\pi d^{4}}$ (4),

where:

$\mu$ – dynamic viscosity [Pa s],

$L$ – segment length [m],

$d$ – segment diameter [m];

For nonlinear model the method of successive approximations was used. The initial parameters of the system, i.e., the arterial resistances of the CoW and attached resistances, as well the initial flow rates, were taken from the linear model. Successive approximations of the values of the nonlinear resistances were calculated from the empirical formulas (2) and (3) and the autoregulatory characteristics, allowing the calculation of successive values of flow rates and pressures. After each iteration, the convergence of the solution was monitored, and the simulation was stopped when a specified level of convergence was reached.
